# Uncovering sex differences of rodent microglia

**DOI:** 10.1186/s12974-021-02124-z

**Published:** 2021-03-17

**Authors:** Jinming Han, Yueshan Fan, Kai Zhou, Klas Blomgren, Robert A. Harris

**Affiliations:** 1 Applied Immunology and Immunotherapy, Department of Clinical Neuroscience, Karolinska Institutet, Center for Molecular Medicine, Karolinska University Hospital, CMM L8:04, Karolinska Sjukhuset, S-171 76 Stockholm, Sweden; 2 Department of Neurosurgery, Tianjin Medical University General Hospital, Tianjin, China; 3 Key Laboratory of Injuries, Variations and Regeneration of Nervous System, Tianjin Neurological Institute, Tianjin, China; 4 Tianjin Medical University, Tianjin, China; 5 Department of Women’s and Children’s Health, Karolinska Institutet, Stockholm, Sweden; 6 Department of Pediatrics, Children’s Hospital of Zhengzhou, Zhengzhou, China; 7 Pediatric Oncology, Karolinska University Hospital, Stockholm, Sweden

**Keywords:** Microglia, Sex differences, Epigenetics, Disease

## Abstract

There are inherent structural and functional differences in the central nervous systems (CNS) of females and males. It has been gradually established that these sex-specific differences are due to a spectrum of genetic, epigenetic, and hormonal factors which actively contribute to the differential incidences, disease courses, and even outcomes of CNS diseases between sexes. Microglia, as principle resident macrophages in the CNS, play a crucial role in both CNS physiology and pathology. However, sex differences of microglia have been relatively unexplored until recently. Emerging data has convincingly demonstrated the existence of sex-dependent structural and functional differences of rodent microglia, consequently changing our current understanding of these versatile cells. In this review, we attempt to comprehensively outline the current advances revealing microglial sex differences in rodent and their potential implications for specific CNS diseases with a stark sex difference. A detailed understanding of molecular processes underlying microglial sex differences is of major importance in design of translational sex- and microglia-specific therapeutic approaches.

## Exploring the sex differences of microglia: a developing story

Every cell has a sex [1]. Neurons and astrocytes in the CNS exhibit obvious sex differences in terms of volume, cell number, and even functional features [2]. However, little attention has been paid to biological sex differences of microglia and associated functions in the neuroimmune system. Microglia have critical effects on CNS development such as contributing to sex differentiation of the brain [3, 4]. They have emerged as central players and promising targets in neurological diseases [5–7], since a spectrum of brain pathologies secondary to neuroinflammation are mainly induced by activated microglia [8, 9]. Males and females may even experience adverse treatment outcomes following specific microglial intervention [10].

Microglia can be reprogrammed during early life and thus exert crucial effects on behavior and disease in later life [11–13]. We reach the realization that CNS diseases developing early in life may be more common in males, while females are most likely to suffer from CNS disorders that emerge later in life [14]. Despite these marked differences, sex has been considered sparsely when making treatment decisions in clinical practice, which might affect the variability of clinical outcomes [15]. Multiple lines of evidence point to dysfunctional glial and neuronal functions as one possible factor that explains sex differences in CNS disease [16]. Sex steroid hormone exposure during critical periods may also exert crucial effects on the functions of several immune cell types [17, 18]. In an attempt to understand such sex differences of microglia, the critical question is when and how microglia become different between males and females?

In this review manuscript, we first summarize recent novel evidence showing the sex differences of rodent microglia. We then discuss potential differences of microglial numbers and phenotypes between sexes. More importantly, microglial endogenous functionality and response to exogenous stimuli also differ between males and females. Epigenetic mechanisms including DNA methylation, histone modifications, and long non-coding RNAs that may partly explain these sex differences of rodent microglia are discussed.

## One hundred years of microglial biology

First described a century ago, microglia are specialized resident macrophages within the central nervous system (CNS) [19–22]. The relatively long-lived microglia are uniquely positioned within the brain parenchyma, colonized from early embryogenesis [23], self-renewed by proliferation throughout adult life [24, 25], underwent four distinct phases of differentiation during development [26], are identified by unique markers [27–29], and are epigenetically primed by early stimulations [30]. Importantly, they are quite distinct from other tissue macrophage populations.

Reports that describe the substantial plasticity and complexity of microglia through a spatiotemporal manner are readily accumulating [31–33]. They are the architects for CNS development and are crucially involved in engulfing cell debris, modulating synaptic elimination by complement [34], axonal migration, and remodeling neural circuits [35, 36]. Apart from their well-appreciated immunological roles, newly emerging microglial functions have been discovered including assisting in oligodendrocyte progenitor maintenance and inducing adult hippocampal neurogenesis [37, 38]. In addition, microglia and astrocytes coordinate their functions via secreted mediators and complement activation, which is fundamental to neuronal functions [39–41].

It is gratifying that most of Hortega’s original postulates of microglial biology have now been proven quite correct, despite that this has required the most recent technical innovations to unequivocally prove his foresight. The most recent findings of spatiotemporal differences between resident microglia subpopulations open up a new era of scientific discovery in which the individual contributions of microglia subsets in both driving or protecting from disease will be possible to define.

## Sex bias in neurological diseases

Sex should be considered as a biological variable when designing both preclinical experiments and clinical trials [42, 43]. Indeed, many neurological disorders demonstrate a sex bias, with neurodevelopmental diseases exhibiting a male bias in incidence [44], while neuroinflammatory diseases such as multiple sclerosis (MS) are female dominated [45]. Taking MS as an example, a recent Danish study demonstrated that the incidence of late onset MS has increased profoundly in women over the previous 60 years but has only slightly increased in men [46]. When compared with male MS patients, female patients have a higher relapse rate, more radiological contrast-enhancing lesions, and stronger adaptive immune activity [45]. However, male MS patients may have more obvious evidence of neurodegeneration, supported by faster brain atrophy and more severe cognitive decline than is experienced by female patients [45].

Colony-stimulating factor 1 receptor (*CSF1R*)-related leukoencephalopathy, mainly caused by *CSF1R* gene mutations and regarded as the most common type of adult-onset leukoencephalopathy, currently without a cure, typically presents with progressive neuropsychiatric and motor symptoms [47, 48]. CSF1R is predominately expressed on microglia within the CNS and thus *CSF1R*-related leukoencephalopathy is considered as a primary CNS microgliopathy with dystrophic microglia playing a pivotal role in disease pathogenesis [47, 49]. Although there are no sex differences of *CSF1R*-related leukoencephalopathy in prevalence and disease duration, male patients develop disease significantly later than do females [50].

Sexual dimorphism of microglial function in other disease conditions was reviewed elsewhere [4, 51]. Given the commonality of the concept, further investigation of other neurological disease settings is warranted.

## Sex differences of microglial morphology and numbers through development

**Fig. 1:**
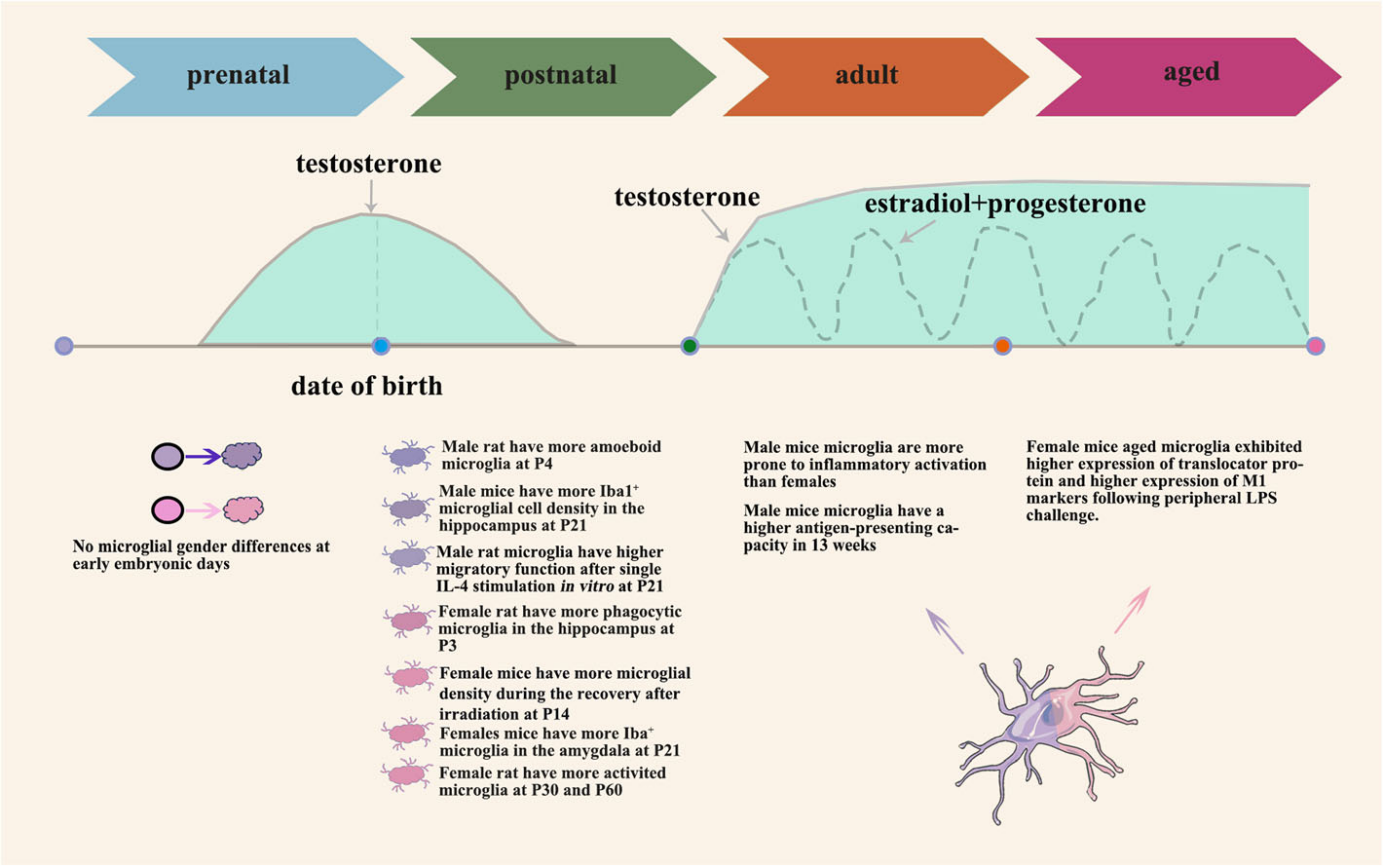
Sex-specific differences of microglia during development and whole life span. Microglia initially colonize the brain during embryogenesis when obvious sex differences of microglia are not apparent. Estradiol and progesterone fluctuations are noted. During the postnatal period, sex hormones such as testosterone can actively influence both the number and function of microglia between males and females. Sex differences of microglia may still exist even later in life. Male microglia (blue) and female microglia (pink)

The current evidence obtained from preclinical models indicates that the number and phenotype of microglia differ between females and males in a region- and age-specific manner (Fig. 1) [52]. The cortex, amygdala, hippocampus, and preoptic area (POA) are well-accepted regions in which obvious sex differences of microglial numbers have been noted [52–55] (Table 1).

**Table 1:** Overview of studies comparing the microglial numbers between males and females in diverse brain regions

Species	Age	Brain region	Main differences	References
Rat	P4	Cortex	Males have more amoeboid microglia	[55]
Rat	P4	Hippocampus	Males have more amoeboid microglia	[55]
Rat	P4	Amygdala	Males have more amoeboid microglia	[55]
Rat	P30	Cortex	Females have more activated microglia	[55]
Rat	P30	Hippocampus	Females have more activated microglia	[55]
Rat	P30	Amygdala	Females have more activated microglia	[55]
Rat	P60	Cortex	Females have more activated microglia	[55]
Rat	P60	Hippocampus	Females have more activated microglia	[55]
Rat	P60	Amygdala	Females have more activated microglia	[55]
Rat	P2	Preoptic area	Males have more amoeboid-shaped microglia	[56]
Rat	P3	Hippocampus	Females have more phagocytic microglia	[53]
Mice	13 weeks	Hippocampus	Males have more Iba1+ microglial cell density	[52]
Mice	13 weeks	Cortex	Males have more Iba1+ microglial cell density	[52]
Mice	13 weeks	Amygdala	Males have more Iba1+ microglial cell density	[52]
Mice	3 weeks	Hippocampus	Males have more Iba1+ microglial cell density	[52]
Mice	3 weeks	Amygdala	Females have more Iba1+ microglial cell density	[52]

*Abbreviations*: *P2* postnatal day 2, *Iba1* Ionized calcium binding adaptor molecule 1

During normal neurodevelopment, microglia undergo sex-specific and distinct maturation processes over time [57]. There are no significant differences in brain region-specific volume and microglial numbers between male and female rats at early embryonic day 17 (E17) [55]. The unique microglial developmental index based on transcriptomic profiling of purified mouse microglia throughout development was also similar between males and females at E18 [57]. Sex differentiation may start after E18 in rodents and extends into the early postnatal period [58]. During postnatal days 2–3 (P2-3), phagocytic hippocampal Iba1^+^ microglia (the presence of at least one phagocytic cup) are significantly more numerous in female rats than in males [53]. In general, exogenous estradiol enhances humoral immunity while testosterone has the opposite effect [15]. In research settings, the number of female rat phagocytic microglia can be reduced to that of male levels following treatment with estradiol during the early postnatal period, indicating a hormonal role for microglial sex differences during rodent brain development [53].

Around the time of birth, a testosterone surge has been noted in male rodents that sensitively influences the number and function of microglia in the developing brain, since microglia expressed related receptors for steroid hormones [58, 59]. In support of this, in the developing POA, a brain region associated with expression of sex behavior and high production of prostaglandin in males, male rat pups have more numerous activated/amoeboid microglia while female rat pups had fewer Iba1^+^-stained microglia on P2 [56].

The sex differences of microglia described above could be mediated by sex hormones, as evidenced by an increased number of amoeboid microglia in the POA in females treated neonatally with estradiol [56, 58]. Furthermore, apparent microglial differences in microglia between males and females have also been noted on P4, shortly after the testosterone surge, when male rats have a more amoeboid microglia than do females in the cortex, hippocampus, and amygdala which diminishes quickly at P30 [55].

As measured using deep single-cell RNA sequencing across different developmental periods and brain regions, adult microglia do not seem as complex and heterogeneous as do postnatal microglia [60]. Nonetheless, microglial sex differences may also be evident in adult rodents (Table 1). Microglia can be influenced by sex identity postnatally and maintained until adulthood independently of circulating sex hormones. Specifically, Iba1^+^ microglial cell density is significantly higher in the hippocampus, cortex, and amygdala of adult male mice when compared with adult female mice [52]. Microglia actually undergo four sequential distinct phases across development based on gene expression [26]. In this case, the microglial morphology, number, and cytokine expression can be modified dramatically throughout neurodevelopment. Indeed, on P30 and P60, rat microglial numbers had opposite trends between males and females in diverse brain regions [55]. It is currently unclear if more microglial progenitor cells may be recruited into the male brain during early development (E8 to E9) and whether more microglial proliferation or less microglial apoptosis occurs in male rodents within a given brain region throughout their life span than in females [4, 24, 61].

We can conclude that microglial sex differences in both space and time are significant in developmental processes and that greater understanding of the underlying molecular mechanisms and environmental cues involved could be vital to the design of tailored therapies.

## Sex differences of microglial functions

Despite the presence of conflicting evidence, differences between rodent male and female comprehensive microglial functionality throughout the life span have also been recently discerned (Table 2), indicating that microglia may not respond similarly between sexes in disease conditions including ischemic stroke and obesity [62–64]. From a functional perspective, microglial phagocytosis is an integral aspect of synaptogenesis and synaptic pruning during fetal development and in adults is an active process necessary to remove dead and dying neurons. Conversely, dysfunctional microglial phagocytosis may contribute to CNS disorders [69]. Interestingly, the upregulation of autophagy via several routes has been shown to promote microglial polarization toward an anti-inflammatory phenotype, while *Atg5* siRNA inhibited autophagy and aggravated proinflammatory polarization [70]. In addition, activation of microglia can also be tightly regulated by the cholinergic pathway through α7 nicotinic receptors [71]. Sex differences of microglial endogenous functions, microglial responses following radiotherapy, and exogenous stimuli will now be discussed.

**Table 2:** Overview of studies comparing the functional differences between male and female microglia

Species	Age	Main functional differences	References
C57BL/6 mice and NF-κB-luc2 mice	12 weeks	Male microglia express more NF-κB regulated genes and are more prone to inflammatory activation than are females. These differences can also be maintained in vitro and thus do not totally depend on sex steroids. Females can even maintain sex-specific microglial differences when transplanted into the opposite sex brain, and this protects the males from ischemic stroke.	[62]
Csf1R-EGFP mice	13 weeks	Sex differences of electrophysiological responses to ATP which measured as membrane currents were noted in isolated microglia. Male microglia have a higher antigen-presenting capacity compared to females. The expression of MHCI was higher in male microglia in both the cortex and hippocampus, while MHCII expression was higher in male cortical microglia.	[52]
C57BL/6 mice	13 weeks	Transcriptional profile differences were found between isolated male and female microglia in both the hippocampus and cortex. Sex differences of microglial steady-state protein levels were also noted from the whole brain.	[52]
CD1 mice	E3,5; adolescent and adult	Treatment of CSF1R inhibitor to deplete embryonic microglia cause sex-specific effects on mice, evidenced by adolescent female mice showing hyperactive development and adult female mice showing anxiolytic-like behavior. However, these phenomena were not noted in male adolescent and adult mice respectively.	[10]
C57BL6/J mice CX3CR1^gfp/gfp^ knock-in mice	18 weeks	There are sex differences of hypothalamic microglial CX3CR1 signaling activation which contribute to obesity susceptibility between male and female mice. Female mice are more resistant to diet-induced obesity than are males. In contrast, female mice become susceptible to diet-induced obesity in the absence of CX3CR1 signaling.	[63]
Sprague Dawley rats	60–90 days	Rat female microglia in the periaqueductal gray area exhibited a more activated phenotype at baseline, produced higher transcription levels of IL-1β, and could be more responsive to immune challenges such as LPS than were male rats, without overall microglial gender density differences in this region.	[64]
Wistar rats	Newborn (P0) to P2	Cultured female rat newborn microglia had higher phagocytic activity than in males as measured by in vitro bead intake assays at both baseline and following by IFNγ stimulation. In contrast, female rat newborn microglia had less basal and stimulated microglial migration than the males as measured by in vitro Transwell assay.	[16]
Sprague Dawley rats	Neonatal	During early postnatal development, phagocytic female microglia in the hippocampus had significantly more highly expressed phagocytic pathway genes and phagocytic associated functions when compared to the males.	[53]
C57BL/6 SPF and GF mice	E18.5 P60	Microglia exert a sex-specific effect on long-term absence of the microbiome, with males being significantly affected during early development while females exhibited profound changes in adulthood instead.	[26]
Sprague Dawley rats	Around 70 days old	Acute or chronic behavioral stress has distinct direct effects on corticolimbic microglial morphology and immune factor transcriptional expression such as CD40, CX3CR1, and CD200R in a number of brain regions which is mediated by microglia in a sex-dependent manner.	[65]
Sprague Dawley rats	Around 70 days old	Differential effects of stress on microglial cell activation in male and female medial prefrontal cortex	[66]
Sprague Dawley rats	P1 and P21	Cultured rat male microglia had increased migration compared to females after single IL-4 stimulation. The mRNA level of K^+^ channel (Kcna3) and Kv current were also higher in male P21 microglia than in females after inflammatory stimulation, while unstimulated microglia had similar levels in males and females.	[67]
C57BI/6J mice	17–18 months	Female aged microglia exhibited higher expression of translocator protein and higher expression of M1 markers following peripheral LPS challenge than did male aged microglia.	[68]

*Abbreviations*: *NF-κB* nuclear factor kappa-light-chain-enhancer of activated B cells, *Csf1R* colony-stimulating factor 1 receptor, *ATP* adenosine triphosphate, *MHC* major histocompatibility complex, *E3,5* embryonic day 3.5, *CX3CR1* CX3C chemokine receptor 1, *LPS* lipopolysaccharide, *IL-1β* interleukin-1beta, *IFNγ* interferon-γ, *GF* germ-free, *SPF* specific-pathogen free

## Sex differences of microglial endogenous functions

Male and female microglia have distinct fundamental endogenous behaviors such as antigen presentation. Some microglial sex differences are noted at both RNA and protein levels with few differentially expressed genes and proteins overlapping [52]. Male microglia exhibit higher potential antigen presentation ability in the cortex, as evidenced by higher expression of major histocompatibility complex (MHC) I and MHC II than do age-matched female microglia [52]. Kinetical observations have demonstrated that microglia are the first local antigen-presenting cells in the CNS to respond and take up myelin antigen, while dendritic cells are more important later in the context of EAE [72]. A possible differential role of microglia during early disease periods may thus be enacted in males and females. This hypothesis is further supported by a recent study elegantly showing that microglia exert a sex-specific effect on the long-term absence of the microbiome, with males being significantly affected during early development while females showed profound changes at adult periods instead [26].

## Sex differences of microglia in response to radiotherapy

Radiotherapy is an effective tool in the treatment of high-grade brain tumors, but it is associated with adverse side-effects, where the long-term, so-called late effects are more pronounced in children [73, 74]. One common such late effect after cranial radiotherapy is cognitive impairment, and this is, at least partly, thought to be caused by reduced hippocampal neurogenesis, where neuroinflammation and a perturbed microenvironment are thought to be involved [75]. We have previously shown that irradiation to the brains of P14 mice can cause in females a more marked upregulation of several cytokines and chemokines in the hippocampus 6 h after irradiation [76]. During recovery, microglial density was increased in females, but not in males, indicating long-lasting effects of irradiation on the hippocampal microenvironment [76]. The subsequent loss of neurogenic capacity and performance in cognitive tests was more pronounced in females [76, 77]. Interestingly, exposure to lipopolysaccharide (LPS) prior to irradiation aggravated the loss of neurogenesis more so in males than in females [76]. Further immuno-radiobiological investigations are needed to understand why girls develop more pronounced cognitive late effects than do boys after cranial radiotherapy [78–81].

## Sex differences of microglia in response to exogenous stimuli

Either acute or chronic behavioral stress has distinct direct effects on corticolimbic microglial morphology and immune factor transcriptional expression, such as of CD40, CX3CR1, and CD200R in a number of brain regions in a microglial sex-specific manner [65, 66]. In addition, cultured rat male microglia had greater migration function than did female microglia following IL-4 stimulation [67], implying that male microglia might have the ability to surround a lesion earlier after brain injury than can females [67, 82]. The mRNA level of K^+^ channel (Kcna3) and Kv current were also higher in male P21 microglia than in females following inflammatory stimulation, while unstimulated microglia had similar levels between males and females [67]. It has been documented that estrogen receptors contribute to mediating sex differences of microglia, as evidenced by perinatal activation of estrogen receptor-α being restricted to males during early development [83]. Overall, such differences may subsequently lead to sex-dependent changes and susceptibility to CNS diseases.

Sex differences in brain innate immune responses to neonatal ischemia have been reported in P12 mice, with higher microglia immunoreactivity, greater proinflammatory gene expression, and increased myeloid cells being noted in male mice [84]. In addition, male neonatal microglia had a significantly greater IL-1β response to LPS compared to neonatal females [85]. Aged mice microglia also exhibited sex differences of immune responses in the context of disease, as evidenced by female aged microglia having higher levels of microglial activation and higher expression of M1 markers following peripheral stimulation than did male aged microglia [68]. However, it is important to note that these microglial sex differences may already exist at this time point before disease induction and may also have a role in differences in secondary damage between males and females. Starting from P60, male microglial maturation is delayed relative to females, and such notable sex differences in microglial maturity may also contribute to disease susceptibility [57]. Specifically, following an acute stimulation such as LPS challenge, an obvious increased level of microglial transcriptional maturation was only observed in males [57]. It has been claimed that male microglia may be more vulnerable to inflammatory stimuli and to be over-activated than in females during a particularly sensitive time window when males have more microglia. This could be a contributory factor to sex differences in both behavior and disease [15, 57].

Male and female microglia may have distinct mechanisms to resolve an inflammatory insult. Specifically, female rat neonatal microglia had both less basal and stimulated microglial migration than did male microglia [16]. During early postnatal development, phagocytic microglia in the female rat hippocampus express significantly more phagocytic pathway genes and phagocytic associated functions when compared to males [53]. In support of this notion, cultured female rat newborn microglia had higher phagocytic activity than males as assessed by in vitro bead uptake assays at both baseline and following interferon (IFN)-γ stimulation [16]. The accumulated data strongly suggest that rat microglia may exhibit sex differences of phagocytic capacity during development [14]. One recent study inferred the opposite conclusion that microglia in male adult mice had similar phagocytic ability as females as measured by in situ phagocytosis assays in both hippocampal and cortical slices [52].

A series of factors such as different ages, species, brain regions, and methods in these studies may account for this discrepancy [86]. In research settings, microglia have to be dissociated from the CNS into a single-cell suspension using both mechanical dissociation and enzymatic dissociation in order to facilitate culture or analyses. Some may argue that mechanical dissociation and enzymatic dissociation may alter the microglial activation status and mRNA expression profiles [87, 88]. Microglia may lose their coordinated cell-to-cell communications when isolated from other CNS cell types, complicating appreciation of their precise roles in vivo [89]. For these reasons, one must be careful in interpreting in vitro studies regarding functional microglial sex differences purported in vivo. Further investigations using advanced methods such as live imaging techniques are warranted to uncover related mechanisms, taking sex differences of microglial phagocytosis into consideration.

Microchimerism is defined as the phenomenon of two genetically distinct cell populations in an individual, with male microchimerism occurring more frequently than in the female brain [90]. Fetal microchimeric cells, genetically foreign maternal cells, persist throughout postnatal development into adulthood, and these microchimeric cells may play a role in responding to injury [90, 91]. Therefore, sexual microglial dimorphism mediated by microchimeric cell effects should not be ignored.

All these observed functional differences may predispose as yet underestimated but marked sex-dependent microglial activation patterns and signaling cascades in the injured CNS [54, 92]. Emerging data has challenged the classical viewpoint that sexual microglial dimorphism is solely due to hormones. The X chromosome inactivation is a main epigenetic feature in females that equalizes dosage of gene products [93]. However, incomplete X chromosome inactivation may cause some genes to be doubly expressed, which could explain specific sex biases [94]. Furthermore, microglial functional balance can be affected by a diversity of environmental factors including stress, with females exhibiting increased stress sensitivity [95]. These non-hormonal mechanisms might also affect microglial development and function. It has been proposed that chronic pregnancy stress and inflammation can be mediated via microglial innate immune memory and that such memory may increase the susceptibility of neurodegenerative conditions later in life [11].

Most of the evidence presented is derived from preclinical studies, showing dynamic sex differences of microglia. Understanding how sex differences of microglia contribute to the occurrence and progression of human CNS disorders may explain sex differences in CNS disease susceptibility and provide new optimism for developing sex-specific treatments.

## Epigenetic control of microglia: novel potential players for sex differences of microglia

Microglia can be pre-conditioned through epigenetic-driven innate immune memory that persists for several months [30, 96]. Epigenetic alterations of microglia during early life may re-program the communication between microglia and other cells in the CNS and have significant later consequences for behavior and disease [11, 13, 61]. We now know that differential use of glycolytic or fatty acid metabolic pathways indices different myeloid cell activation states [97] and so differential microglial functionality between males and females might be due to underlying metabolic differences. Epigenetic processes can integrate intrinsic signals and environmental variables into the genome and then regulate microglial genes and functions that contribute to the sex bias in autoimmune diseases [45, 98]. Microglial differentiation and response to stimuli can be governed by epigenetic mechanisms including DNA methylation, histone modifications, and long non-coding RNAs [99].

DNA methylation is an epigenetic mechanism that occurs by the addition of methyl groups to DNA, modifying genetic functions without changing the sequence [96]. From a sex viewpoint, females have been reported to have higher levels of DNA methyltransferase enzyme activity, DNA methylation, and methylated CpG sites than do males in the neonatal POA [100]. Inhibiting DNA methyltransferase causes masculinized neuronal markers and male sexual behavior in female rats [100], suggesting that DNA methylation serves as the basis for the sex-specific transcriptomes in selective brain regions.

Histone modifications are epigenetic regulators that control chromatin structure and gene expression [101]. Histone modifications associated with neural sexual differentiation were recorded [102]. Epigenetic regulation of microglia may vary across life span, prenatal depletion of the histone deacetylases Hadc1 and Hadc2 using *Cx3cr1*^Cre^Hdac1^fl/fl^Hdac2^fl/fl^ mice being harmful for microglial development by inducing apoptosis and reducing survival [103, 104]. However, experimental depletion of the histone deacetylases Hadc1 and Hadc2 during adulthood did not impair microglial numbers and morphology, indicating that epigenetic factors may not be critical for adult steady-state microglia [103, 104]. While no sex differences in histone modifications were evident in whole embryonic brains [102], regionally the acetylation and methylation of histone H3 in neonatal male mice were increased in the cortex and hippocampus when compared with females, but not in the POA, amygdala, and hypothalamus [102].

MicroRNAs are small non-coding RNA molecules that act as post-transcriptional negative regulators of gene expression. Different expressions of isolated microglial microRNAs in the adult mice brain between male and female at baseline have also been uncovered using microRNA sequencing, which was also validated using qPCR [105]. Selective loss of these microRNAs by ablating the microRNA-processing enzyme Dicer lead to significant sex-dependent changes of microglial transcriptomes with male adult microglia exhibiting a higher extent than the females [105]. Furthermore, male and female mice showed a similar Tau burden in P301S tauopathy mice, while sex-related changes of microRNA profiles were evident in the condition of tau pathology, suggesting that microglial microRNAs may serve as a potential player for mediating sex differences of microglia [105]. In support of this, certain microRNA can modulate acetylcholine signaling by regulating cholinergic gene expression [106]. Of relevance, dysregulated acetylcholine signaling can lead to CNS and peripheral dysfunctions via a sex-specific manner [106, 107], potentially shedding light onto other neuroinflammatory diseases.

Collectively, these data imply that epigenetic mechanisms such as DNA methylation, histone modifications, and microRNAs impact an array of transcriptional responses and, at least in part, represent a potential mechanism that might explain microglial sex-related functional differences. We are optimistic that future research regarding epigenetic control of male and female microglia will bear exciting novel discoveries to better understand the sex differences of microglia.

## Conclusions

Sex differences of microglia exist among diverse regions with respect to both CNS physiology and pathology. Sexual microglial dimorphism may be in part responsible for sex differences in the incidence and pathology of a variety of neurological diseases. Males may be more vulnerable to neurological insults during early development in part as a result of having more highly activated microglia in the developing brain compared to females. In contrast, increased activation of microglia in females during adulthood may contribute to their increased susceptibility to several inflammatory brain diseases that occur late in life. Given our increasing understanding of these fascinating cells in some degree of both targets and drivers of sex differentiation [108], future investigation will bear exciting novel discoveries about sex microglia. Sex differences of microglia could be caused by intrinsic differences evidenced by X chromosome containing a large density of immune-related genes and some epigenetic modifiers, while the differences can also be mediated by hormonal or other environmental influences expressed over the lifespan. Therefore, more effort will be needed to explore how genes located on the X or Y chromosomes, epigenetic mechanisms, endocrine factors, and microenvironmental signals synergistically contribute to microglial sex differences and then fully understand the molecular bias of CNS diseases with sex vulnerability [108, 109].

## Data Availability

Not applicable.
